# A case report of a patient with ductal carcinoma and a malignant phyllodes tumor in situ in 2 separate breasts

**DOI:** 10.1097/MD.0000000000036405

**Published:** 2023-12-01

**Authors:** Chen Li, Chun Zhang

**Affiliations:** a Department of Breast Surgery, Peking University International Hospital, Beijing, China.

**Keywords:** breast, ductal carcinoma in situ, malignant phyllodes tumor

## Abstract

**Rationale:**

Breast malignant phyllodes tumors (MPT) are quite uncommon. It is rarely reported that they occur in conjunction with breast cancer. We detailed a case in which an MPT and ductal carcinoma in situ carcinoma occurred simultaneously in 2 different breasts.

**Patient concerns:**

A 79-year-old female patient was seen for a rapidly growing lump in the upper left quadrant of her breast. The lump was described as huge, hard, irregular, and palpable. MRI of the breasts revealed a big mass in the left breast and a smaller lump in the right.

**Diagnosis:**

Ductal carcinoma in situ with breast MPT.

**Interventions:**

We performed a double mastectomy. Post-operative endocrine treatment was suggested.

**Outcomes:**

During the 18-month follow-up period, no signs of recurrence or metastasis were seen. The ultrasound examination of the chest wall showed no abnormality. Bilateral axillary and supraclavicular ultrasonography showed no lymphadenectasis and a CT scan of the lungs showed no suspicious cancer nodules.

**Lessons:**

It is possible for MPT and ductal carcinoma in situ to occur simultaneously in different breasts. Surgeons need to integrate clinical observations, imaging tools, and patient history to make an early diagnosis. Before undergoing surgery, a thorough examination of both breasts is required.

## 1. Introduction

Breast phyllodes tumors (PT) are uncommon biphasic neoplasms made up of epithelial and stromal parts.^[1]^ They account for 2% to 3% of all breast fibrous epithelial tumors and 0.3% to 1.0% of all breast cancers, respectively.^[2,3]^ PT is categorized as benign, borderline, or malignant based on the presence or absence of cellular atypia, stromal pleomorphism, mitotic index, stromal expansion, tumor margin, and heterologous differentiation. Breast cancer and any kind of PT may occur simultaneously in a single or opposite breast.^[4–6]^ It is unusual for malignant PT (MPT) to develop in 1 breast while ductal carcinoma in situ develops in the other at the same time. We report a case in which an MPT and ductal carcinoma in situ carcinoma occurred simultaneously in 2 different breasts. Written informed consent was obtained from the patient and her daughter for publication of this case report and any accompanying images.

## 2. Case report

A 79-year-old lady noticed a rapidly expanding lump in the upper left quadrant of her breast. The lump was huge, hard, irregular, and palpable. The skin around the nipple and the areola were both healthy. No history of breast cancer in her family was recorded. She was admitted to our breast surgery ward in March 2022.

**Figure 1.**
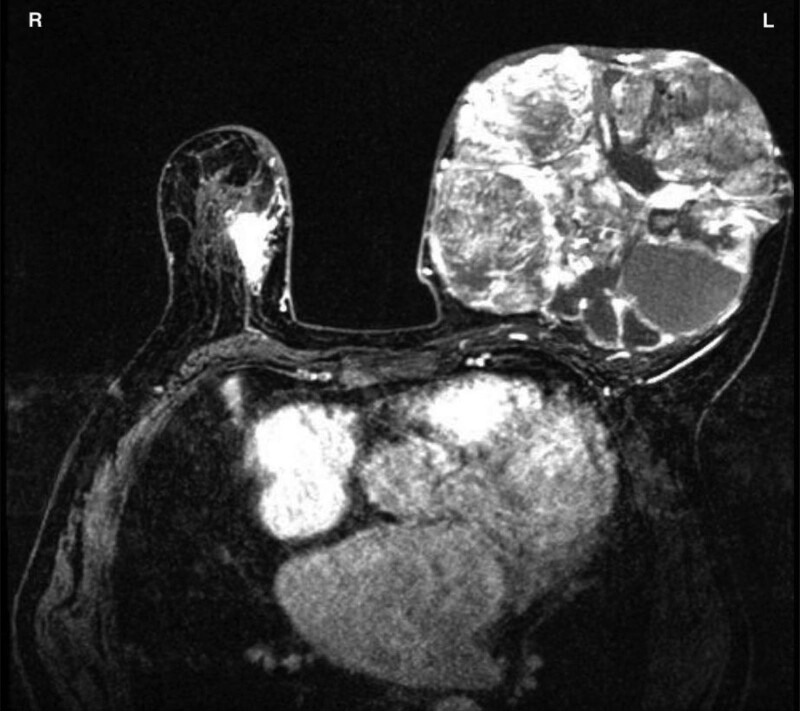
MRI of the breasts revealed a big mass in the left breast and a smaller lump in the right.

On admission, magnetic resonance imaging of the breasts revealed a 13.4 cm tumor in the left breast and a 2.8 cm mass in the right (Fig. 1). Biopsies taken with a core needle revealed ductal carcinoma in situ on the right breast and PT on the left. A biopsy of the sentinel lymph node in the right axilla yielded negative pathological results. Chest and abdominal computed tomography scans revealed no new abnormalities. No skeletal metastases were seen on a comprehensive bone scan.

**Figure 2.**
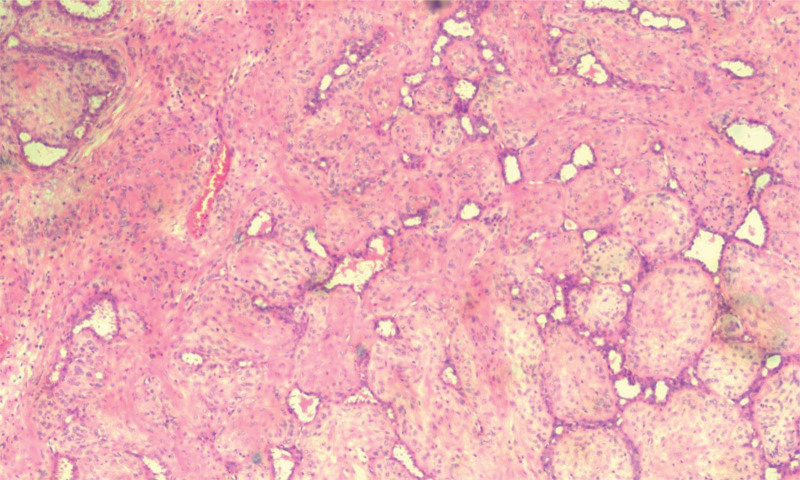
Histological results of malignant phyllodes tumor.

**Figure 3.**
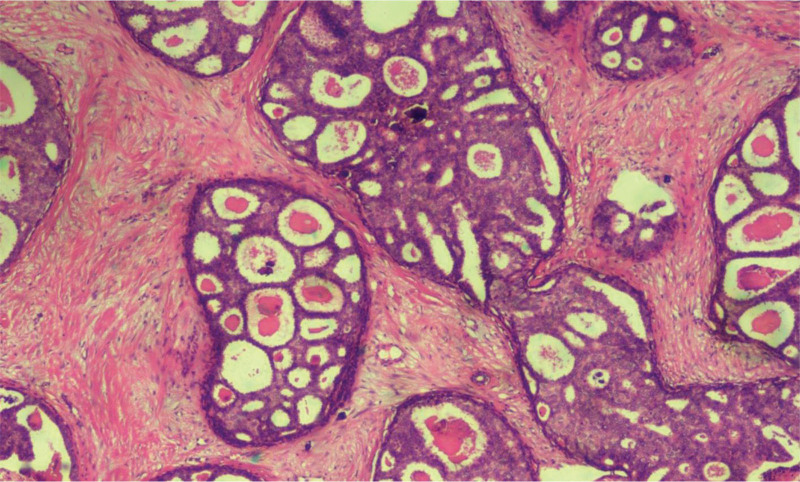
Histological results of low to intermediate grade ductal carcinoma in situ carcinoma.

After about 2 weeks, we opted on a bilateral mastectomy after considering the diagnosis and the magnitude of the lesions. After surgery, the left breast was found to have a big, uneven, partially nodular lesion covered by an elliptical skin that was 15 × 12 × 12 cm, gray-white, lobulated, and myxoid in appearance. One nodule, 4 × 2 × 1.5 cm, was found in the right breast. Malignant PT was confirmed by anatomic and pathological examination of the patient left breast. The tumor displayed pleomorphism and mitoses (10–12/10HPF), as well as cartilaginous and osseous metaplasia (Fig. 2). Figure 3 shows that the right breast contained a low- to intermediate-grade ductal carcinoma in situ carcinoma. Immunohistochemical analysis revealed that ductal carcinoma in situ carcinoma was highly positive for progesterone and estrogen receptors but negative (score 0) for human epidermal growth factor receptor 2. After discharge, the patient began endocrine treatment.

After 18 months of follow-up, there have been no noticeable changes in the patient clinical status or test results as of yet. The ultrasound examination of the chest wall showed no abnormality. Bilateral axillary and supraclavicular ultrasonography showed no lymphadenectasis and a CT scan of the lungs showed no suspicious cancer nodules.

## 3. Discussion

This case study highlights the possibility of MPT and ductal carcinoma in situ carcinoma occurring simultaneously in 2 different breasts.

Based on their pathological characteristics, PT were termed by the international histological classification group of the World Health Organization in 1981 and classified as benign, borderline, or malignant.^[7]^ Typical PT measure between 4 and 5 cm in length, whereas MPT may go quite a bit bigger than that. They typically develop gradually over time but may experience explosive expansion suddenly. PT are usually solitary, located on one side of the body, have a nodular appearance, cause no discomfort, and may be of a surprisingly tiny or enormous size. In clinical practice, a PT may be recommended if the patient has a history of fast growth, is tall for their age, and/or is very enormous in size.^[8]^

PT of the breast is a unique subset of mammary neoplasms that occurs very seldom. This means that both stromal and epithelial components are present simultaneously, making them biphasic. The degree of stromal differentiation is predictive of malignancy. In very unusual cases, the MPT may coexist with cancer. Rare instances of MPT with coexisting breast cancer in the contralateral breast were described.^[4–6,9–11]^ There are 2 patterns to the coexistence of PT and breast cancer: distinct coexistence inside an ipsilateral or contralateral breast, and a breast cancer originating within PT. How this works on a biological level is a mystery and a point of debate. Here, we detail the example of a woman who had simultaneous diagnoses of MPT and bilateral breast cancer.

It is unclear what mechanisms link PT to breast cancer. The development of a PT and the coexistence of breast cancer inside the PT have both been linked to genetic alterations with intratumoral heterogeneity.^[12]^ This transition of epithelial and mesenchymal components may be triggered by a variety of factors, including a mutation in the p53 gene. However, this does not provide light on the PT-independent etiology of breast cancer. It is possible that the link is coincidental, but we still need to learn more about the genetics involved. In contrast to the more benign PT, the aggressive clinical characteristics, tendency for local recurrence, and capability for distant metastasis of MPT make it almost specific to this disease. Hematogenous metastasis often affects many organs, including the lungs and bones.^[13–15]^ Rarely can metastases from MPT spread to the axillary lymph nodes.

More than 8% of cases in 10 years showed local recurrence in benign forms; this number is much higher in borderline and malignant forms.^[16]^ In addition, studies have shown that 22% to 50% of MPT cases return locally following breast-conserving surgery with a negative margin.^[17]^ Tumors that are very large or numerous, as well as those with nuclear atypia, tumor necrosis, or pleomorphism, are all thought to increase the likelihood of a local recurrence.^[18,19]^ Despite their rarity, MPTs tend to have favorable survival rates. Cause-specific survival rates for MPT were predicted to be 92.2% for 5 years, 90.8% for ten years, and 90.3% for 15 years.^[20]^ However, metastasis almost often means increased risk of death.^[21,22]^

Treatment for MPT has traditionally focused on surgical intervention. Surgical therapy-wide excision without axillary staging has been indicated as the primary treatment for MPT of the breast by the National Comprehensive Cancer Network Guideline. There is the option of a complete mastectomy or breast-conserving surgery with clean margins of more than 10 mm. Since metastases to the axillary lymph nodes are uncommon, axillary dissection was not done routinely.^[23,24]^ However, the debate over mastectomy vs breast-conserving surgery continues to this day. Cases with MPT should be selectively treated according to tumor size, according to a review of the Surveillance, Epidemiology, and End Results 1902 data (2000–2009). Patients whose tumors are ≤ 10 cm in diameter may benefit more from breast-conserving surgery.^[25]^ However, a number of researchers have shown that MPT dramatically reduces the recurrence incidence of breast cancer in women who have had mastectomy.^[26]^ Recurrence and disease-free survival were not improved by removing lymph nodes from the armpit in patients with MPT, according to the majority of studies.^[8,27–30]^

There is some disagreement over whether or not radiation has a role in treating MPT. The relative risk of local recurrence in cases managed with postoperative radiation treatment was reduced compared to patients not treated with postoperative radiation therapy, even in the setting of margin-negative extensive local excision, as revealed in a meta-analysis.^[31]^ Radiation treatment was shown to reduce local recurrence in breast-conserving surgery patients, according to a review of the National Cancer Data Base.^[32]^ Breast-conserving surgery with a negative margin has been shown to be effective against MPT, however, other studies have indicated that postoperative radiation treatment is necessary.^[14,20]^ The 5-year disease-free survival rates of patients who had breast-conserving surgery with radiation treatment (tumor-free margin < 1 cm) or breast-conserving surgery alone (tumor-free margin ≥ 1 cm) were similar, according to a single study.^[33]^ Radiation treatment was associated with worse cancer-specific survival in a Surveillance, Epidemiology, and End Results study of patient data from 1983 through 2002.^[34]^ Although no local recurrence was seen in patients who had adjuvant radiation therapy, the effectiveness of this treatment is still debatable, as reported by Park et al^[18]^

The general consensus among clinicians was that patients at high risk or with a positive resection margin would benefit from more aggressive therapy. More research is required to determine the best course of action for these individuals. Due to the lack of high-risk characteristics and the presence of these contradictory pieces of information, we did not conduct radiotherapy.

Due to a paucity of information from big prospective trials, we do not yet know how effective adjuvant chemotherapy is in PT treatment. There is more work to be done in determining chemotherapy function in both the adjuvant and palliative contexts. Adjuvant chemotherapy after surgical treatment may be warranted as a suitable rationale for the malignancy of the epithelial component, despite the fact that chemotherapy is often reserved for metastatic cases in MPT.^[35,36]^ There was no evidence of distant metastases in our imaging tests. The patient did not have adjuvant chemotherapy since she was diagnosed with ductal carcinoma in situ cancer of the right breast. Endocrine therapy was used as adjuvant treatment for ductal carcinoma in situ of the right breast. During the 18-month follow-up period, no signs of metastasis or recurrence were seen.

## 4. Limitations of the study

There are some limitations of the current study. First, although we referenced the National Comprehensive Cancer Network guidelines, our treatment decisions may not have been optimal because of the limited literature and high-level evidence available for this case. However, the results of the 18-month follow-up showed that our treatment decision could be considered for this rare case. Second, longer-term follow-up results are currently lacking. However, the follow-up of the patient is still ongoing. Third, as with most of the case reports, there are methodological limitations in terms of the retrospective nature of the report and the fact that it lacks the power for generalization.

## 5. Conclusions

Independent and simultaneous lesions in both breasts are an extremely unusual manifestation of MPT-associated breast cancer. A thorough examination of both breasts before surgery is required to aid in early diagnosis. Surgeons must integrate data from patient records, imaging studies, and clinical examinations. Our example illustrates the significance of considering the possibility of axillary lymph node examination and subsequent adjuvant treatment when diagnosing PT. To maximize the clinical care of these malignancies when they coexist in early identification and management, a rigorous assessment of real-world data is necessary.

## Author contributions

**Conceptualization:** Chen Li.

**Data curation:** Chen Li.

**Formal analysis:** Chen Li.

**Investigation:** Chen Li.

**Resources:** Chen Li.

**Writing – original draft:** Chen Li.

**Writing – review & editing:** Chun Zhang.
